# Supplementary material for: Stress, subjective wellbeing and self-knowledge in higher education teachers: A pilot study through bodyfulness approaches
Source: PLoS One. 2022 Dec 15;17(12):e0278372. doi: 10.1371/journal.pone.0278372 (PMC9754221; doi:10.1371/journal.pone.0278372)
Supplement: S1 Appendix — (DOCX) [file pone.0278372.s001.docx]

**S1. Supporting Information Appendix 1**

**Procedure to translate to Spanish the Body Awareness Questionnaire (BAQ)** (Shields, Mallory & Simon, 1989).

The original BAQ English language instrument was translated into Spanish independently by one Spanish native speaker with intensive English language training and knowledge of the English-speaking culture, and an English native speaker, teacher of English and with high level of Spanish language (Dr. Cristina Bamond). The translation aimed at a conceptual equivalent of the respective item rather than a word-for-word translation. Both translations were combined by the translators into a single consensus translation by discussion. The instrument was then back-translated into English by an independent professional who had no knowledge of the original instrument and had no health background. Again, a single back-translation was produced by discussion until consensus was reached. Concordance of the back-translated version and the original BAQ was discussed by the translators, the developer of the original instrument (SS) and an English native speaking (Dr. Carolina Pulido). This discussion resulted in slightly changing item 18, “Percibo reacciones corporales específicas cuando estoy muy hambriento”, more closely reflecting the original wording over-hungry.
