# Supplementary material for: Stress, subjective wellbeing and self-knowledge in higher education teachers: A pilot study through bodyfulness approaches
Source: PLoS One. 2022 Dec 15;17(12):e0278372. doi: 10.1371/journal.pone.0278372 (PMC9754221; doi:10.1371/journal.pone.0278372)
Supplement: S2 Appendix — (DOCX) [file pone.0278372.s002.docx]

**S2. Supporting Information Appendix 2**

**Requirements and limitations for participants saliva collection for the determination of cortisol level**

The participants should not have done high intense exercise on the morning of the sampling day and should not have eaten anything in the 45 minutes prior to taking the sample. Teachers were asked about any abnormal events that might have occurred on the sampling day that could modify the cortisol level. Saliva samples had to be completely transparent. Those samples that showed the slightest red color were discarded because blood contamination falsely elevates the actual cortisol content. When this happened, the participant was instructed to wash his mouth with cold water and waited 10 minutes before collecting the saliva sample again. It was not allowed to chew anything during the sampling process in order to generate more saliva. Instead, they could drink water to stimulate saliva production but then had to wait 5 minutes to collect the sample.
