# Supplementary material for: Stress, subjective wellbeing and self-knowledge in higher education teachers: A pilot study through bodyfulness approaches
Source: PLoS One. 2022 Dec 15;17(12):e0278372. doi: 10.1371/journal.pone.0278372 (PMC9754221; doi:10.1371/journal.pone.0278372)
Supplement: S3 Appendix — ANOVA post intervention and paired-sample t-test pre and post intervention for all the variables and groups. (DOCX) [file pone.0278372.s003.docx]

**S3. Supporting Information Appendix 3**

**Additional statistical data analysis. ANOVA post intervention and paired-sample t-test pre and post intervention for all the variables and groups.**

**Mean, standard deviation and results of ANOVA test for the three groups (Control, HY and BMA) and all the variables after intervention**

| **Variables** | **Control (n=10)** | **HY (n=11)** | **BMA (n=10)** | **p-value^*^** |
| --- | --- | --- | --- | --- |
| Body Awareness (BAQ) | 85,18 ± 15,21 | 89,89 ± 18,59 | 87,45 ± 14,51 | 0,809 |
| Mindfulness (FFMQ) | 130,50 ± 14,63 | 129,78 ± 14,38 | 133,50 ± 17,39 | 0,856 |
| Subjective wellbeing (WHO-5) | 62,40 ± 20,15 | 66,67 ± 14,83 | 65,78 ± 16,51 | 0,766 |
| Life satisfaction (SWLS) | 27,56 ± 2,01 | 28,00 ± 3,08 | 29,22 ± 2,39 | 0,367 |
| Perceived stress (PSS) | 19,10 ± 5,09 (a) (b) | 12,13 ± 6,96 (a) | 11,38 ± 4,78 (b) | 0,013 |
| Sleep efficiency (%) | 81,90 ± 12,55 | 87,16 ± 5,90 | 85,17 ± 7,62 | 0,455 |
| Low frequency (LF, nu) ^**^ | 58,12 ± 17,76 | 65,04 ± 13,70 | 57,11 ± 23,58 | 0,591 |
| High frequency (HF, nu)^**^ | 41,75 ± 17,65 | 34,89 ± 13,66 | 42,72 ± 23,54 | 0,596457 |
| Ratio LF/HF (ms^2^) | 2,11 ± 2,01 | 2,67 ± 2,06 | 1,14 ± 0,92 | 0,724 |
| Cortisol value | 3,39 ± 1,10 | 2,97 ± 1,07 | 2,90 ± 0,81 | 0,757 |
| ^*^significative differences (p<0,05) among the three groups  ^**^Low frequency (LF) and high frequency (HF) are normalized components of heart rate variability | | | | |

| **Results of paired-sample t-test pre- and post-intervention for all the variables and control, HY and BMA groups**   \|  \| Control (n=10) \| \| \| \| HY (n=11) \| \| \| \| BMA (n=10) \| \| \| \| \| --- \| --- \| --- \| --- \| --- \| --- \| --- \| --- \| --- \| --- \| --- \| --- \| --- \| \|  \| Pre  (mean ± SD) \| Post  (mean ± SD) \| p-value* \| Cohen's d \| Pre  (mean ± SD) \| Post  (mean ± SD) \| p-value* \| Cohen's d \| Pre  (mean ± SD) \| Post  (mean ± SD) \| p-value* \| Cohen's d \| \| Body Awareness** \| 81,14 ± 19,67 \| 87,14 ± 14,24 \| 0,115 \| 0,697 \| 75,50 ± 20,25 \| 88,88 ± 19,61 \| **0,000** \| 4,063 \| 75,36 ± 22,17 \| 87,45 ± 14,51 \| 0,061 \| 0,636 \| \| Changes in Body Processes \| 25,86 ± 5,15 \| 28,28 ± 6,97 \| 0,035 \| 1,025 \| 24,38 ± 9,10 \| 29,25 ± 8,08 \| **0,016** \| 1,119 \| 23,91 ± 7,61 \| 28,09 ± 4,72 \| 0,101 \| 0,545 \| \| Body Reactions \| 27,43 ± 8,40 \| 32,43 ± 5,19 \| 0,047 \| 0,939 \| 28,00 ± 8,67 \| 32,13 ± 8,76 \| **0,003** \| 1,561 \| 27,64 ± 8,92 \| 33,36 ± 6,89 \| **0,024** \| 0,802 \| \| Sleep-Wake Cycle \| 29,71 ± 7,61 \| 30,86 ± 4,67 \| 0,558 \| 0,234 \| 26,88 ± 7,66 \| 31,00 ± 7,01 \| **0,009** \| 1,261 \| 26,82 ± 9,49 \| 30,45 ± 4,91 \| 0,166 \| 0,451 \| \| Onset of Illness \| 19,57 ± 5,96 \| 18,57 ± 4,69 \| 0,197 \| 0,548 \| 16,88 ± 2,80 \| 20,25 ± 4,13 \| **0,012** \| 1,195 \| 16,91 ± 6,33 \| 19,09 ± 4,06 \| 0,351 \| 0,295 \| \| Mindfulness \| 127,00 ± 19,98 \| 131,25 ± 16,39 \| 0,423 \| 0,301 \| 124,38 ± 12,72 \| 130,75 ± 15,05 \| 0,252 \| 0,441 \| 130,60 ± 18,45 \| 133,50 ± 17,39 \| 0,148 \| 0,500 \| \| Subjective wellbeing \| 59,50 ±19,88 \| 58,00 ± 17,24 \| 0,747 \| 0,119 \| 56,5 ± 18,07 \| 66,00 ± 15,71 \| **0,008** \| 1,286 \| 64,44 ± 12,40 \| 65,78 ± 16,51 \| 0,790 \| 0,092 \| \| Life satisfaction \| 24,43 ± 2,94 \| 26,86 ± 1,57 \| 0,051 \| 0,921 \| 26,25 ± 4,74 \| 27,63 ± 3,07 \| 0,120 \| 0,625 \| 27,33 ± 2,92 \| 29,22 ± 2,39 \| 0,051 \| 0,764 \| \| Perceived stress \| 19,14 ± 7,54 \| 20,00 ± 5,77 \| 0,656 \| 0,177 \| 17,88 ± 6,56 \| 12,13 ± 6,96 \| **0,012** \| 1,190 \| 10,63 ± 2,92 \| 11,38 ± 4,78 \| 0,706 \| 0,139 \| \| Sleep efficiency (%) \| 84,98 ± 5,20 \| 81,90 ± 12,55 \| 0,493 \| 0,215 \| 83,34 ± 7,44 \| 87,16 ± 5,90 \| 0,181 \| 0,488 \| 87,95 ± 4,79 \| 85,17 ± 7,62 \| 0,380 \| 0,292 \| \| Low frequency (nu) \| 54,95 ± 14,45 \| 58,12 ± 17,76 \| 0,666 \| 0,134 \| 52,90 ± 14,99 \| 65,04 ± 13,70 \| 0,064 \| 0,669 \| 46,17 ± 19,47 \| 57,11 ± 23,58 \| 0,087 \| 0,608 \| \| High frequency (nu) \| 44,94 ± 14,38 \| 41,75 ± 17,65 \| 0,662 \| 0,136 \| 47,07 ± 14,99 \| 34,89 ± 13,66 \| 0,063 \| 0,672 \| 53,72 ± 19,51 \| 42,72 ± 23,54 \| 0,082 \| 0,619 \| \| Low / High frequency (ms2) \| 1,55± 0,37 \| 2,11 ± 0,60 \| 0,451 \| 0,236 \| 1,55 ± 0,52 \| 2,67 ± 0,87 \| **0,039** \| 0,762 \| 1,14 ± 0,29 \| 2,27 ± 2,32 \| 0,093 \| 0,594 \| \| Cortisol \| 2,97 ± 0,80 \| 3,39 ± 1,10 \| 0,206 \| 0,408 \| 3,0 ± 1,1 \| 2,32 ± 0,82 \| 0,549 \| 0,197 \| 3,66 ± 1,62 \| 2,90 ± 0,83 \| 0,292 \| 0,402 \|   ^*^significative differences (p<0,05); ^**^four scales for Body Awareness are included: changes in body processes; body reactions; sleep-wake cycle and onset of illness |
| --- | --- | --- | --- | --- | --- | --- | --- | --- | --- | --- | --- | --- | --- | --- | --- | --- | --- | --- | --- | --- | --- | --- | --- | --- | --- | --- | --- | --- | --- | --- | --- | --- | --- | --- | --- | --- | --- | --- | --- | --- | --- | --- | --- | --- | --- | --- | --- | --- | --- | --- | --- | --- | --- | --- | --- | --- | --- | --- | --- | --- | --- | --- | --- | --- | --- | --- | --- | --- | --- | --- | --- | --- | --- | --- | --- | --- | --- | --- | --- | --- | --- | --- | --- | --- | --- | --- | --- | --- | --- | --- | --- | --- | --- | --- | --- | --- | --- | --- | --- | --- | --- | --- | --- | --- | --- | --- | --- | --- | --- | --- | --- | --- | --- | --- | --- | --- | --- | --- | --- | --- | --- | --- | --- | --- | --- | --- | --- | --- | --- | --- | --- | --- | --- | --- | --- | --- | --- | --- | --- | --- | --- | --- | --- | --- | --- | --- | --- | --- | --- | --- | --- | --- | --- | --- | --- | --- | --- | --- | --- | --- | --- | --- | --- | --- | --- | --- | --- | --- | --- | --- | --- | --- | --- | --- | --- | --- | --- | --- | --- | --- | --- | --- | --- | --- | --- | --- | --- | --- | --- | --- | --- | --- | --- | --- | --- | --- | --- | --- | --- | --- | --- | --- | --- | --- | --- | --- | --- | --- |
