# Supplementary material for: Stress, subjective wellbeing and self-knowledge in higher education teachers: A pilot study through bodyfulness approaches
Source: PLoS One. 2022 Dec 15;17(12):e0278372. doi: 10.1371/journal.pone.0278372 (PMC9754221; doi:10.1371/journal.pone.0278372)
Supplement: Attachment — Submitted filename: COMMENTS.docx [file pone.0278372.s004.docx]

The current study has adopted a mixed method approach – using experimental data and content analysis – to study the efficacy of two intervention on stress, subjective well-being, and self knowledge of higher education teachers. The authors have wisely opted for mixed method considering the limitation of the study with only quantitative assessment, but effort is needed to combine the outcomes of quantitative and qualitative domains and present it as complimentary to each other. Although the attempt was elaborate with many variables, the study apparently has many limitations that need to be addressed before publication. Especially the variables described at different parts of the manuscript are not congruent. Eg. The study measures listed contain FFMQ, to assess mindfulness, but nowhere in the manuscript the authors have mentioned about mindfulness. Similarly, the variables measured and variables described as the outcomes are not the same. Further comments are added here.

**Title**: Why there are two titles given (1-6)?

**Abstract**: Wordiness can be reduced in the abstract. Eg. in lines 20-22, the word ‘theirs’ is repeated five times, which is unnecessary.

Clearly state the number of groups, ie. two experimental groups and one control group with number of participants in each.

**Introduction**: In line 57, please include the in-text citation apart from the numbering.

**Materials and Methods**: lines 163-166 – Please explain what PARQ+ did assess. “participants should 166 not have lengthy practice with any of the tools proposed in the program” – was this another exclusion criterion? (Then better to mention exclusion *criteria*) Does it mean that the participants should not already have lengthy practices? Some more clarity is required here as what the authors have meant, since the intervention is not elaborated in the manuscript so far. Also please define lengthy. What was the duration beyond which a practice was considered lengthy?

Lines 171-173: please mention the number of participants in all three groups. You may write “Group 1 (N=?).

Line 175: instead vaguely mentioning “*several* physiological variables”, please clearly describe all the physiological variables assessed.

Lines 177-179: “The levels of body awareness, subjective wellbeing and perceived stress level were measured using five validated psychometric tests…”. Why five assessment tools were used to assess three variables mentioned? Also pleas explain why there are seven psychological assessment tools listed under ‘Measures’ and not five? Also the measures described in lines 219-285 do not correspond to the variables mentioned in line 177-179. Eg. FFMQ was administered, but assessment of mindfulness is not given in the variables listed in lines 177-179.

Line 181: did the authors mean “Participants *filled*….” Also, five questionnaires? Please explain why seven are listed under Measures.

Line 230: Couldn’t find the full form of MET. If you haven’t called it out (mentioning the full form along with acronym at the first usage of an acronym) before, please do so.

The selection procedures for the recruitment of participants to the experimental condition can be represented using a CONSORT flow diagram. Were there any dropouts? And why?

The recruitment process of participants for the qualitative part of the study is not clear. Also please describe more about the need and procedures of qualitative data collection.

Please include a brief description of the administration of both interventions including who have administered both and if the differences in expertise could be a reason for some of the results obtained.

**Results**:

Lines 356-357: “None of the potential participants who responded to the PARQ+ were excluded from the study”. Why? Also please clearly describe the outcomes of any assessment tools you have used to screen the participants (as exclusion criteria).

Results require substantial changes.

Other than the figures, please include tables containing:

1) Comparison of baseline scores of all three groups on all variables under study. You can use ANOVA, if the data fulfill the parametric test requirements. At least for the variables where it is normally distributed, please report ANOVA results. Also, if the data is not normally distributed, please explain the possible reasons.
2) Comparison of post-test scores of all three groups on all variables under study. Here also, ANOVA should be the first option.
3) T-test result comparing the pre-post results of all variables for all three groups. Eg. Pre-post comparison of mindfulness scores of experimental Group 1.

31 individuals have participated in the quantitative part of the study, but only 22 are there in the qualitative part. Please explain why with the selection procedure.

**Discussion**:

Line 673 says sample size was 33, but earlier it was given as 31 for quantitative and 22 for qualitative. Please clarify.

Line 688: the meaning of the sub-title is not clear.

Please include the limitations of the study.

**Conclusion**:

“In our opinion, a combination of both programs could provide a safe framework that would help to increase the levels of teacher wellbeing and give them resources to improve interaction with students, given that in education the quality of interpersonal relationships is an important part of the teaching-learning process”. Both the interventions share many common components as displayed in a table earlier. So what exactly did the authors mean by the application of both program together? Are you proposing a novel intervention? Have you considered the possibility of the outcomes measures of the current study and the outcomes of the interventions being different, thus producing not much significant results?

Another major issue is that, since there already are many highly effective interventions to reduce stress and enhance well-being, what is the purpose of these new bodyfulness interventions? Even the study results do not support much significant impact. So how do the authors justify its contribution to the existing literature?
