# Supplementary material for: Stress, subjective wellbeing and self-knowledge in higher education teachers: A pilot study through bodyfulness approaches
Source: PLoS One. 2022 Dec 15;17(12):e0278372. doi: 10.1371/journal.pone.0278372 (PMC9754221; doi:10.1371/journal.pone.0278372)
Supplement: Attachment — Submitted filename: Response to reviewers.docx [file pone.0278372.s005.docx]

We greatly appreciate the time taken to review the article and the suggestions made. Responses are indicated in this document in red and changes to the manuscript and additional material are provided in accompanying documents.

Reviewer #1: It was a pleasure reading the authors’ work and in their detailed description of their intervention. However, the manuscript requires further re-writing.
First of all, the authors need to provide a shorter and better structured Discussion section. Currently the section is more than 10-pages long and parts of it may be suitable for a dissertation chapter but offer little value to the discussion of the findings of their paper. For example, their opening paragraphs (lines 669-687) in their Discussion focus on their methodology rationale that would be a better bit to their methodology section. Similarly, the first few pages in the Discussion they go on to be a presentation of methodology-related relevant literature that may again be a good fit to a dissertation’s methodology chapter but it offers little to the Discussion of the research findings. My advice to the authors is to reduce significantly the length of that section. They can start with a couple of paragraphs discussing their research objectives and methodology decisions within the scope of relevant literature and the move on to discussing their findings in relation to other studies in teachers or application of these interventions in other populations

We have re-written the discussion section following reviewer’s indications.
The title of the manuscript mentions that this is a “proposal of intervention”, however their methodology, and results section describe what may be best defined as a “pilot” study. If the authors indeed aimed to report the development of an intervention, then they’d need t restructure the paper to clearly show what parts are relevant to the “development” of the intervention and what elements involve its “delivery”.

We intended to carry out a pilot study, so we have included some modifications to clarify this point in the title, abstract, introduction and methodology (lines 27-31,155, 170-179 and 191-200). Also some modifications have been introduced in methodology and results looking for more clarity.

Reviewer #2: The current study has adopted a mixed method approach – using experimental data and content analysis – to study the efficacy of two intervention on stress, subjective well-being, and self-knowledge of higher education teachers. The authors have wisely opted for mixed method considering the limitation of the study with only quantitative assessment, but effort is needed to combine the outcomes of quantitative and qualitative domains and present it as complimentary to each other. Although the attempt was elaborate with many variables, the study apparently has many limitations that need to be addressed before publication. Especially the variables described at different parts of the manuscript are not congruent. Eg. The study measures listed contain FFMQ, to assess mindfulness, but nowhere in the manuscript the authors have mentioned about mindfulness. Similarly, the variables measured and variables described as the outcomes are not the same.

We have better explained the variables and the reasons why a mixed approach was chosen (see lines 192-202; lines 246-2502)

Further comments are added here.

Title: Why there are two titles given (1-6)?

The title was adjusted to the requirements of this journal. It is the journal format.

Abstract: Wordiness can be reduced in the abstract. Eg. in lines 20-22, the word ‘theirs’ is repeated five times, which is unnecessary.

We have rewritten the abstract according the reviewer’s suggestions.

Clearly state the number of groups, ie. two experimental groups and one control group with number of participants in each.

Done

Introduction: In line 57, please include the in-text citation apart from the numbering.

Done (see line 62 now)

Materials and Methods: lines 163-166 – Please explain what PARQ+ did assess. “participants should 166 not have lengthy practice with any of the tools proposed in the program” – was this another exclusion criterion? (Then better to mention exclusion criteria) Does it mean that the participants should not already have lengthy practices? Some more clarity is required here as what the authors have meant, since the intervention is not elaborated in the manuscript so far. Also please define lengthy. What was the duration beyond which a practice was considered lengthy?

We have tried to explain better according reviewer’s suggestion (see lines 170-172). PARQ + is a 7-step self-screening tool that is typically used to determine the safety or possible risks of exercising based on the health history, current symptoms, and risk factors. It is designed to identify the small number of adults for whom physical activity may be inappropriate. If a person answers yes to one or more questions, the individual should complete a thorough follow-up of medical questions and consult a physician about beginning physical activity.

We consider a lengthy practice any physical activity similar to those implemented in the project practiced during at least six months on a weekly basis. Following the reviewer’s suggestion, we have introduced this as an exclusion criterion (see lines 172-174).

Lines 171-173: please mention the number of participants in all three groups. You may write “Group 1 (N=?).

Done (now this information is in lines 181-183).

Line 175: instead vaguely mentioning “several physiological variables”, please clearly describe all the physiological variables assessed.

Done (see now lines 197-199).

Lines 177-179: “The levels of body awareness, subjective wellbeing and perceived stress level were measured using five validated psychometric tests…”. Why five assessment tools were used to assess three variables mentioned? Also pleas explain why there are seven psychological assessment tools listed under ‘Measures’ and not five? Also the measures described in lines 219-285 do not correspond to the variables mentioned in line 177-179. Eg. FFMQ was administered, but assessment of mindfulness is not given in the variables listed in lines 177-179.

We assessed five psychological variables. Following the reviewer’s suggestion we have detailed these five variables in lines 246-252 (previously lines 177-179).

There are no adapted tools for body movement awareness interventions, reason why authors decided to use five validated psychometric tests, as they covered different aspects on which the programs could have an impact.

Line 181: did the authors mean “Participants filled….” Also, five questionnaires? Please explain why seven are listed under Measures.

Participants filled two questionnaires about physical activity. One of them, the PARQ+ was used as exclusion criteria in the recruitment phase. Five questionnaires were used for psychological variables. See lines 246-252.

Line 230: Couldn’t find the full form of MET. If you haven’t called it out (mentioning the full form along with acronym at the first usage of an acronym) before, please do so.

Done. We had included the full form in Figure 1, but in fact we had not included the first usage of the acronym, so we have resolved this including the full form along with acronym (line 264).

The selection procedures for the recruitment of participants to the experimental condition can be represented using a CONSORT flow diagram. Were there any dropouts? And why?

Following the reviewer’s suggestion, we have included a CONSORT flow diagram as Figure 1 showing the recruitment process. Accordingly, we have renumbered all figures.

The recruitment process of participants for the qualitative part of the study is not clear. Also please describe more about the need and procedures of qualitative data collection.

There is no different procedure for recruiting participants for the qualitative part. They are the same participants. A slightly change in line 347 tries to clarify this. We have also included this information in the CONSORT flow diagram.

Please include a brief description of the administration of both interventions including who have administered both and if the differences in expertise could be a reason for some of the results obtained.

We have reinforced the explanation requested about the expertise levels of the professionals in charge of the different programs (lines 218-220). We have also included it as a potential limitation (lines 1035-1039).

Results:
Lines 356-357: “None of the potential participants who responded to the PARQ+ were excluded from the study”. Why? Also please clearly describe the outcomes of any assessment tools you have used to screen the participants (as exclusion criteria).

We have added more details in lines 392-396 (previously lines 356-357). The screening tool requires that the participant answers YES to at least one of the 7 initial questions of the test in order to continue with further questions and suggest a medical check-up before doing any kind of physical activity. This was not the case as all participants answered NO to all 7 initial questions.

Results require substantial changes.

Other than the figures, please include tables containing:
1) Comparison of baseline scores of all three groups on all variables under study. You can use ANOVA, if the data fulfill the parametric test requirements. At least for the variables where it is normally distributed, please report ANOVA results. Also, if the data is not normally distributed, please explain the possible reasons.

We have included the results of ANOVA pre-test in the manuscript as Table 5. We have renumbered the rest of the tables accordingly

2) Comparison of post-test scores of all three groups on all variables under study. Here also, ANOVA should be the first option.

We have included the results of ANOVA post-test as a Supplementary file S3.

3) T-test result comparing the pre-post results of all variables for all three groups. Eg. Pre-post comparison of mindfulness scores of experimental Group 1.
We have included the results of T-test in the Supplementary file S3 to complement the figures and text.

31 individuals have participated in the quantitative part of the study, but only 22 are there in the qualitative part. Please explain why with the selection procedure.

There was a typo mistake in the number. Correct number is 21. Only the participants who participated in the interventions (group 2 and 3, n=11 and n=10, respectively) wrote reflexive diaries (see lines 505-506). We have also added this information in the CONSORT diagram (Figure 1).

Discussion:

Line 673 says sample size was 33, but earlier it was given as 31 for quantitative and 22 for qualitative. Please clarify.

That was a typo mistake. Total number of participants for the three groups were 31. Only the participants who participated in the interventions (group 2 and 3, n=11 and n=10, respectively) wrote reflexive diaries (see lines 505-506). We have clarified this in the text and through a CONSORT diagram (Figure 1).

Line 688: the meaning of the sub-title is not clear.

The sub-title follows the format requirements stablished by the journal.

Please include the limitations of the study.

We have reinforced the limitations of the study in lines 1015-1042.

Conclusion:
“In our opinion, a combination of both programs could provide a safe framework that would help to increase the levels of teacher wellbeing and give them resources to improve interaction with students, given that in education the quality of interpersonal relationships is an important part of the teaching-learning process”. Both the interventions share many common components as displayed in a table earlier. So what exactly did the authors mean by the application of both program together? Are you proposing a novel intervention? Have you considered the possibility of the outcomes measures of the current study and the outcomes of the interventions being different, thus producing not much significant results?

We have modified the text to try to better explain our point of view. We show differences in two mind-body approaches. We believe that a successful intervention could take advantage of the strengths of both approaches by combining resources from both. In fact, we have carried out another study with engineering students in which we combined the sequential structure of Hatha Yoga with creative movement, play and symbolism in a relational context provided by Dance Movement Therapy (<https://doi.org/10.3390/educsci12020111>).

Another major issue is that, since there already are many highly effective interventions to reduce stress and enhance well-being, what is the purpose of these new bodyfulness interventions? Even the study results do not support much significant impact. So how do the authors justify its contribution to the existing literature?

Dance movement therapy is not a new bodyfulness intervention. Its origin is in the 1940 in United States and there are many evidences about its effectivity in mental health. The novelty of this paper lies in its introduction in higher education that until now is scarce. The relational component, not included in others approaches, justify the introduction of this approach in education.
