# Supplementary material for: Stress, subjective wellbeing and self-knowledge in higher education teachers: A pilot study through bodyfulness approaches
Source: PLoS One. 2022 Dec 15;17(12):e0278372. doi: 10.1371/journal.pone.0278372 (PMC9754221; doi:10.1371/journal.pone.0278372)
Supplement: Attachment — Submitted filename: Respond to reviewers.docx [file pone.0278372.s006.docx]

Journal Requirements:
Please review your reference list to ensure that it is complete and correct. If you have cited papers that have been retracted, please include the rationale for doing so in the manuscript text, or remove these references and replace them with relevant current references. Any changes to the reference list should be mentioned in the rebuttal letter that accompanies your revised manuscript. If you need to cite a retracted article, indicate the article’s retracted status in the References list and also include a citation and full reference for the retraction notice.

Additional Editor Comments:
Thank you for submitting a revision of your manuscript. I am the designated Guest Editor to handle this document and I was also part of the first round of the review process. It is clear that the quality of the manuscript has substantially improved and the authors have done a great work addressing the comments raised in the review process. I am satisfied with the authors’ response and revisions to my comments and especially regarding the material that needed to be presented in different sections which allowed a better evaluation of the manuscript.

However, there are still some remaining issues which I believe can be easily amended:

-Currently, the authors do not clearly signpost their outcome measures. Just as the authors explain in their response they used a set of questionnaires to measure specific psychological variables. My recommendation is to rename the title “Measures” (if possible) to “Outcome measures” and the processed to title the scales used as “Quantitative Measures”

Following the editor’s indications, we have renamed the title “Measures” to “Outcome Measures”. We have left the other titles for the quantitative and qualitative measures as they were in order to respect the limitations of the journal’s guidelines to three heading levels. Anyway, we have introduced additional comments in lines 249 and 256 to clarify the type of measures used.

- Is it possible to break-down the study objectives? Currently in line 155 only the aim of the pilot study is provided along with a reference in lines 159-162 to the differences between the two approaches.

Following the editor’s suggestions, we have broken-down the general objectives in two specific objectives (see lines 157-161).

-The main remaining weakness of the paper is that it offers little insight on the necessity of the qualitative data. Were those data meant to address a specific separate research question? If yes, it would be useful to add it at the end of the introduction section. If, though, they are complementary then the authors would need to elaborate a bit on how they are combined.

We have tried to better justify the importance of introducing qualitative data at the end of the introduction. In our view, mixed approaches, combining quantitative and qualitative data, enrich and complement the information gathered by shedding more light on processes, and are essential in areas such as the one we are dealing with. Some references have been added to remark this consideration (see lines 162-169).

-I found very helpful the authors’ link to another piece of their work, within which they mention that they conducted “An experimental pre-post design with a control group was combined with an exploratory qualitative approach”. Does that description also apply to the current study? If yes then I would recommend to rephrase it slightly and use that to describe the current study. Similarly, it would help to separate between “Quantitative measures” and “Qualitative Exploration”.

Yes, that description also apply to this study. We think that we have clarified better the design with the additional information added throughout the new version of the paper.

-In their previous paper that the authors cite in their response to reviewers they mention that “a structured warm up was designed following the characteristics Hatha Yoga Is that an example of combining the two approaches?

Yes, in the paper cited, some elements from the two approaches were combined. In fact, one was the structured Hatha Yoga warm-up. In the present paper, the two warm-ups were different, each one of them following their own criteria adapted to the respective programs.

If yes, then I would recommend adding that argument and possibly pick up on findings from their current study (one example would be enough) on how one could go to combine other elements of those two approaches.

We have tried to include one specific example in the discussion (see lines 880-886).

-The authors report that there were some differences in the effects of the two approaches, which was also evident in their qualitative analysis. It would be useful to read what this could possibly mean for the content of future interventions and their targeted outcomes. I believe that they have pointed towards that that in lines 1041-1044: “Furthermore, the choice of assessment instruments seems to be more appropriate for the HY approach. In future work, it would be interesting to incorporate instruments that capture relational aspects as they are essential in the state of life satisfaction, well-being and self-knowledge of the individual”. For this reason, it would be useful to add a sentence in the discussion commenting on the nature of the instruments they used in this study and possibly highlight that the qualitative exploration signals possibly the need to use in future feasibility study other types of outcome measures.

We have added the idea suggested (see lines 923-927) and rename the heading “limitations” to “limitations and future directions” to be coherent with the content.

-Finally, I would like to pick up on the authors’ response that “The novelty of this paper lies in its introduction in higher education that until now is scarce. The relational component, not included in others approaches, justify the introduction of this approach in education.”

I believe that this is an important statement that should be added at the end of the introduction.

Following the editor’s recommendation, we have included this statement at the end of the introduction (lines 167-169).

Furthermore, I think it would be greatly beneficial if the authors reflect on other factors that may influence or impact on intervention implementation or effectiveness within higher education. Are there any context factors that they have identified that they would need to address in a future feasibility study?

You are right. In fact, contextual factors are really important. We have included this consideration in the discussion, lines 910-921.

Although their intervention did not adopt a mindfulness-based approach, are there lessons from other types of bodyfulness/mindfulness-based interventions applied in higher education that are also relevant to the implementation and evaluation of the type of intervention that the authors describe? Do they differ to what we know about other interventions aiming to improve wellbeing and reduce stress?

We have reinforced the importance of relational component to improve wellbeing and reducing stress. We have also underlined the importance of a structured frame (as mindfulness-based interventions offer) that help participants to increase body awareness, body knowledge and self-confidence regarding the own body (see lines 825-827].
